# Gender differences in cardiovascular outcomes of kidney transplant recipients: A retrospective cohort study

**DOI:** 10.1097/MD.0000000000039568

**Published:** 2024-09-13

**Authors:** Jiang Liu, Siwei Chen, Wenqiang Gao

**Affiliations:** a Department of Cardiovascular Medicine, Yingtan People’s Hospital, Jiangxi, P.R. China; b Department of Cardiovascular Medicine, The Third Hospital of Nanchang, Jiangxi, P.R. China; c Department of Urology, Zaozhuang Municipal Hospital, Zaozhuang, P.R. China.

**Keywords:** end-stage renal disease, kidney transplantation, major adverse cardiovascular events, propensity score matching

## Abstract

The purpose of this study was to investigate gender differences in cardiovascular outcomes of kidney transplant recipients (KTRs). Here, a retrospective cohort study was conducted, and data from the National Health Insurance Research Database in Taiwan were used. In total, 2904 patients who had end-stage renal disease (ERSD) and received kidney transplantation (KT) were identified by propensity score matching (PSM) and were enrolled from 1997 to 2012, with follow-up ending in 2013. Besides, major adverse cardiovascular events (MACEs) were defined as a composite of all-cause mortality, nonfatal myocardial infarction, and nonfatal strokes. Apart from that, hazard ratios (HRs) and 95% confidence intervals (CIs) were calculated by Cox regression, while the Bayesian network model was constructed to assess the importance of risk factors for MACEs. Furthermore, the original cohort was a sensitivity analysis. Women had a lower risk of MACEs compared with men (hazard ratio [HR]: 0.84; 95% CI: 0.72–0.98; *P* = .024). Beyond that, stratified analysis of age and waiting time for KT showed that the risk of MACEs was significantly lower in women than in men among KTRs aged > 50 years (HR: 0.79; 95% CI: 0.62–1.0; *P* = .05) or waiting time for KT ≤ 6 years (HR: 0.85; 95% CI: 0.72–0.99; *P* = .04). Bayesian network indicated that age is an important determinant of cardiovascular outcomes in KTRs, regardless of gender. In Taiwan, women had a lower risk of adverse cardiovascular outcomes than men in KTRs aged > 50 years or with a waiting time for KT ≤ 6 years. Furthermore, age is an important independent determinant for the prognosis of KTRs.

## 1. Introduction

The prevalence and incidence of end-stage renal disease (ESRD) are higher in Asian countries, such as China, Japan, and Korea.^[1]^ Currently, renal replacement therapy, including maintenance dialysis and kidney transplantation (KT) is the primary treatment modality for ERSD.^[2]^ Compared to patients receiving KT, ESRD patients on maintenance dialysis have substantial mortality, with cardiovascular and cerebrovascular events accounting for 50%.^[3]^ Despite advances in patient selection, organ harvesting and preservation, surgical techniques, immunosuppression, and infection prevention that have led to significant improvements in rejection and infection after KT, cardiovascular risk in kidney transplant recipients (KTRs) remains dramatically higher than in the general population.^[4,5]^ Furthermore, cardiovascular disease is still the major cause of premature death in most kidney transplant registries.^[6]^ Due to the shortage of kidney donors, knowledge about the prognosis of KTRs is mainly derived from observational population-based studies, clinical trials, and extrapolation of nontransplantation studies.^[7]^ Multiple previous studies have shown that advanced age, prior cardiovascular disease, and dyslipidemia are independent risk factors for prognosis in KTRs.^[8–11]^ Evidence for gender differences in cardiovascular outcomes of KTRs remains limited.^[12]^ Therefore, the purpose of this study was to investigate gender differences in cardiovascular outcomes of KTRs.

## 2. Methods

### 2.1. Study populations and data sources

Here, a retrospective cohort study was conducted, and data from the National Health Insurance Research Database in Taiwan were used. The detailed study procedure was reported previously.^[2]^ In brief, the National Health Insurance Research Database covered inpatient and outpatient medical data for nearly 23 million residents in Taiwan. All renal replacement therapy strategies, including KT and maintenance dialysis, were recorded by the National Health Insurance (NHI) system, where the patient’s ID number, age, gender, detailed medical records, previous disease history, and diagnosis were also contained.

**Figure 1.**
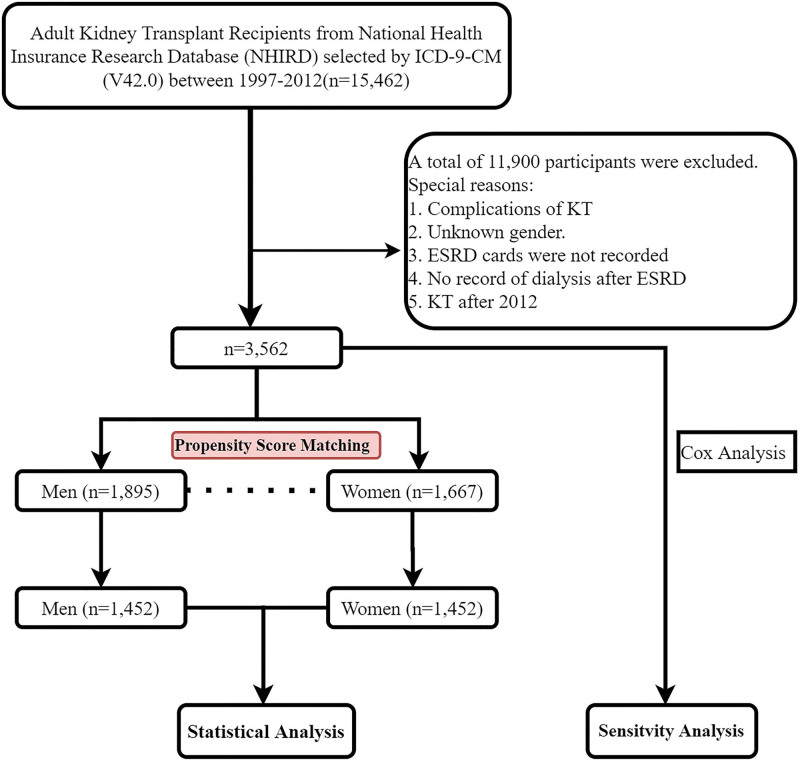
Study flow. ESRD= end-stage renal disease, NHIRD = National Health Insurance Research Database, KT = kidney transplantation.

According to the International Classification of Diseases, 9th Revision, Clinical Modification (ICD-9-CM), 15,462 ESRD patients aged ≥ 18 years in the insurance system were identified from January 1, 1997 to December 31, 2012. After excluding those with unknown ESRD diagnosis and incomplete dialysis information, and those with < 2 years of follow-up, 3562 patients remained. Of these 3562 patients, 1895 were men and 1667 were women. Afterward, the baseline characteristics of men and women were matched according to the propensity matching score (PSM), and finally 1452 cases for men and women separately were included in the statistical analysis (Fig. 1). Besides, the analysis of the full data before matching was the sensitivity analysis. All participants were followed up until the end of December 31, 2013.

This study was in accordance with the Declaration of Helsinki and was approved by the Institutional Review Board of Kaohsiung Veterans General Hospital (VGHKS15-EM10-02). All participant information was anonymous without infringing on their rights. This study was written under the STROBE guidelines.

### 2.2. Definitions

Diagnostic dates for ESRD and KT are defined as the dates recorded on ESRD and KT certificate cards, and the time difference between them was the waiting time for KT. Besides, maintenance dialysis is allowed before and after KT. Furthermore, according to ICD-9-CM, ESRD and KT were coded as 585 and V42.0, respectively.

For the other comorbidities, diabetes mellitus, hypertension, dyslipidemia, prior myocardial infarction (MI), and ischemic strokes were coded as 250.X, 401.X-405.X, 272.X, 410.X-411.X, and 433-434, accordingly.

### 2.3. Outcome definitions

All-cause death was defined as any cause of death that withdraws from the NHI system, while nonfatal MI and nonfatal strokes were coded as 410.X-411.X and 433-434, respectively. Otherwise, the primary outcome was the major adverse cardiovascular events (MACEs), which was a composite of all-cause mortality, nonfatal MI, and nonfatal strokes, whereas secondary outcomes were all-cause mortality, nonfatal MI, and nonfatal strokes, respectively.

### 2.4. Statistical analysis

Categorical variables were expressed as percentages and analyzed by the chi-square test, while continuous variables were analyzed using *t* tests or Mann–Whitney *U* tests, depending on the data distribution. To minimize differences in baseline characteristics between men and women, PSM was adopted to identify patients with similar baseline characteristics. PSM was performed without replacement using a 1:1 protocol with a caliper width of 0.008. Although more stringent calipers have been attempted, the best matching results were obtained with a caliper width of 0.008.

Kaplan–Meier analysis with the log-rank test was used to analyze the cumulative rates of primary and secondary outcomes between men and women during the observation period. Besides, the proportional hazard assumption was tested by Schoenfeld residuals, while univariate and multivariate Cox regression analyses were performed to identify significant parameters associated with primary and secondary outcomes. The results were expressed by hazard ratios (HRs) and 95% confidence intervals (CIs). In addition, following PSM, multivariate Cox regression analyses were performed on variables with *P* values for between-group differences <0.1 to further exclude confounders, and the results were considered sensitivity analyses.

Additionally, a Bayesian network (BN) model was constructed to facilitate our understanding of the relationship between baseline characteristics and the primary outcomes. Specifically, the BN model is a probabilistic graphical model that combines probability theory and graph theory to reveal the probabilistic correlations between variables (nodes). An arrow connecting two nodes indicates that two random variables are causally or unconditionally independent; if there is no arrow connecting two nodes, it is shown that the random variables are conditionally independent.^[13]^ Thus, the probability of an outcome occurring in the presence of multiple conditional variables was inferred, while the importance of the variables was obtained. In the BN model, each circle represents a predictor, and the shade of its color indicates its importance for the occurrence of MACEs, with darker colors denoting higher importance. Other than that, the BN was created in the SPSS Modeler model section based on the Tree Augmented Native (TAN) algorithm, and parametric learning methods were chosen as Bayesian adjustments for small cell counts.^[14]^

All software analyses were performed using the R-based packages, Statsape Software (Rosyclouds Co. Ltd), IBM SPSS (version 26.0; SPSS Inc., Chicago, IL, USA), IBM SPSS Modeler (Version 18.0; IBM Canada Ltd., Markham, Ontario, Canada), STATA (Version 12.0; StataCorporation, College Station, TX, USA), and GraphPad Prism (Version 9.0; USA, San Diego, CA). The two-tailed *P *< .05 indicated statistical significance.

## 3. Results

Before PSM, the original cohort included a total of 3562 individuals with a median follow-up of 7.8 years (interquartile range: 4.9–10.8), of which 1895 and 1667 were men and women, respectively. The data distribution of men and women at baseline characteristics was unbalanced and statistically significant. After PSM, a total of 2904 individuals were matched, 1452 for both men and women, and their data distributions on baseline characteristics were more balanced than before with no statistically significant differences (Table 1 and Figure S1, Supplemental Digital Content, http://links.lww.com/MD/N528).

**Table 1:** Baseline characteristics of men and women in the cohort before and after PSM.

Gender	Before PSM n = 3562		p-value	After PSM n = 2904		*P*-value
	Men n = 1895	Women n = 1667		Men n = 1452	Women n = 1452	
Age			0.013			0.90
≤40 yrs	712 (37.6%)	641 (38.5%)		568 (39.1%)	578 (39.8%)	
41–60 yrs	1042 (55%)	942 (56.5%)		813 (56%)	801 (55.2%)	
>60 yrs	141 (7.4%)	84 (5%)		71 (4.9%)	73 (5%)	
Waiting time for KT						>0.99
<1 yr	491 (25.9%)	362 (21.7%)	<0.001	345 (23.8%)	345 (23.8%)	
1–3 yrs	886 (46.8%)	766 (46.0%)		680 (46.8%)	679 (46.7%)	
4–6 yrs	385 (20.3%)	365 (21.9%)		301 (20.7%)	302 (20.8%)	
≥6 yrs	113 (7%)	174 (10.4%)		126 (8.7%)	126 (8.7%)	
Diabetes			<0.001			0.66
No	1409 (74.4%)	1394 (83.6%)		1207 (83.1%)	1197 (82.4%)	
Yes	486 (25.6%)	273 (16.4%)		245 (16.9%)	255 (17.6%)	
Hypertension			<0.001			>0.99
No	398 (21%)	430 (25.8%)		354 (24.4%)	354 (24.4%)	
Yes	1497 (79%)	1237 (74.2%)		1098 (75.6%)	1098 (75.6%)	
Dyslipidemia						>0.99
No	1323 (69.8%)	1264 (75.8%)	<0.001	1094 (75.3%)	1094 (75.3%)	
Yes	572 (30.2%)	403 (24.2%)		358 (24.7%)	358 (24.7%)	
History of AMI			0.007			0.06
No	1843 (97.3%)	1643 (98.6)		1432 (98.6%)	1443 (99.4%)	
Yes	52 (2.7%)	24 (1.4%)		20 (1.4%)	9 (0.6%)	
History of ischemic stroke			<0.001			>0.99
No	1841 (97.2%)	1650 (99%)		1441 (99.2%)	1440 (99.2%)	
Yes	54 (2.8%)	17 (1%)		11 (0.8%)	12 (0.8%)	

AMI = acute myocardial infraction, KT = Kidney transplantation, PSM = propensity score matching.

### 3.1. Survival Analysis

**Figure 2.**
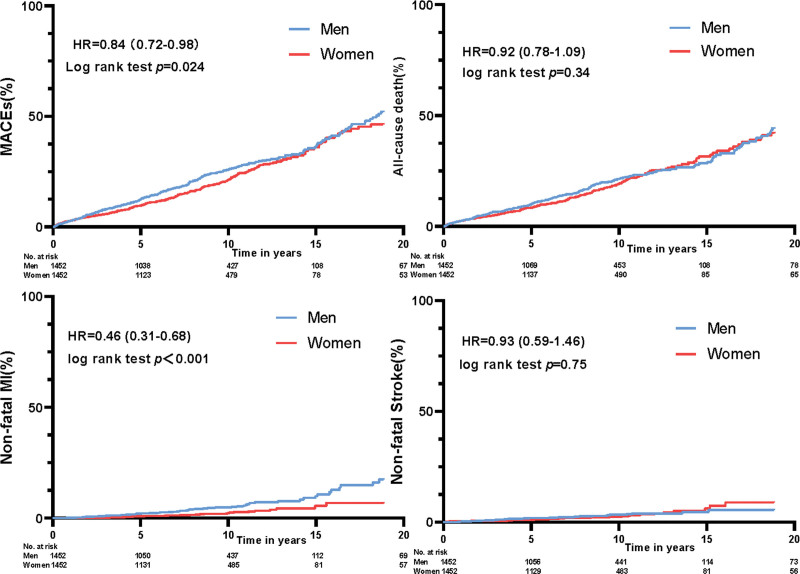
Kaplan–Meier analysis of MACEs, all-cause death, nonfatal MI, and nonfatal stroke among men and women in PSM cohort. HR = hazard ratio, MACE = major adverse cardiovascular event, MI = myocardial infarction, PSM = propensity score matching.

Among the 2954 KTRs included in analysis, 645 developed MACEs occurred during the observational period. All survival curves passed the proportional hazard assumption (Table S1, Supplemental Digital Content, http://links.lww.com/MD/N528. Compared with men, the risk of MACEs in women was lower (HR: 0.84; 95% CI: 0.72–0.98; *P* = .024) (Fig. 2). In the secondary outcomes, women had an obviously lower risk of nonfatal MI than men (HR: 0.46; 95% CI: 0.31–0.68; *P *< .001). However, there was no significant difference in the risk of all-cause death (HR: 0.92; 95% CI: 0.78–1.09; *P* = .34) and nonfatal strokes (HR: 0.93; 95% CI: 0.59–1.46; *P* = .75) between men and women (Fig. 2). As a sensitivity analysis, consistent results were acquired in the original cohort (Figure S2, Supplemental Digital Content, http://links.lww.com/MD/N528). In addition, when the history of prior AMI was adjusted to the multivariate Cox regression model, the results still showed that the risk of MACE was lower in women than in men (HR: 0.84; 95% CI: 0.72–0.99; *P* = .033).

### 3.2. Subgroup analysis and Multivariate cox regression

**Figure 3.**
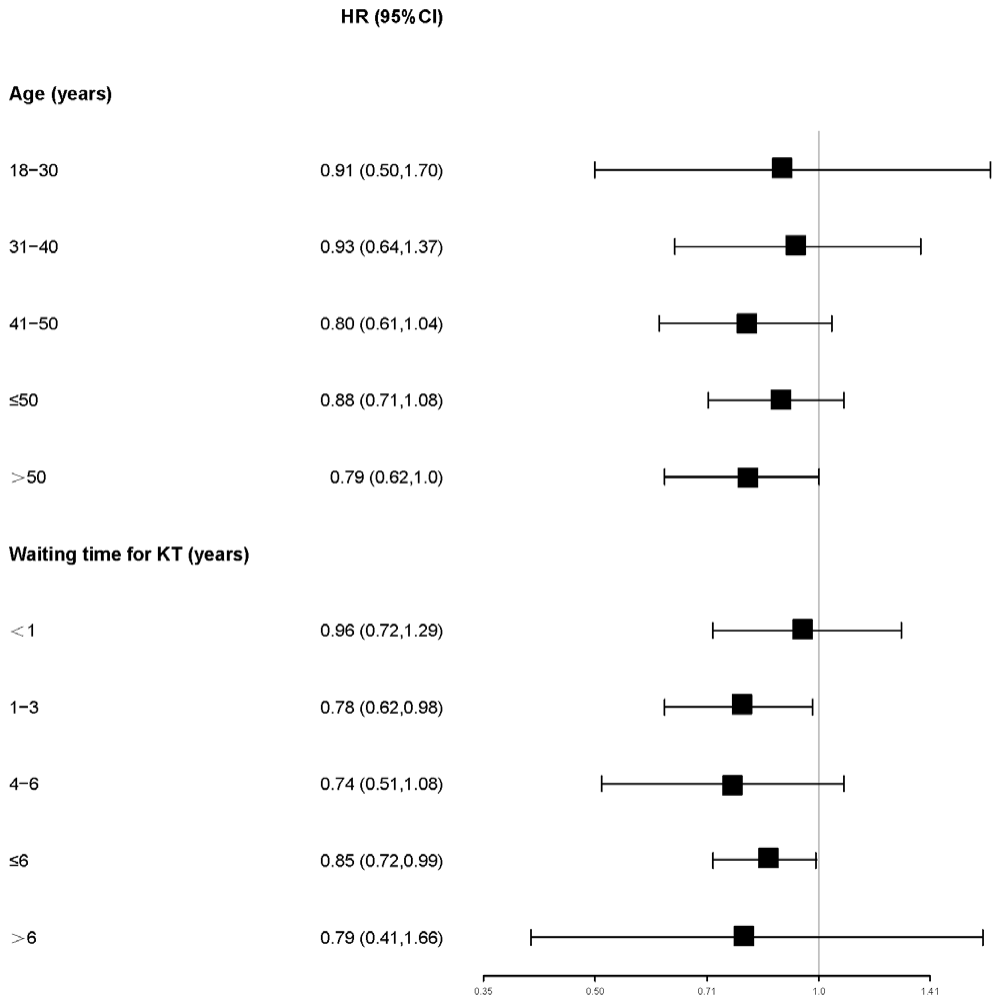
Cox regression analysis of MACE for men and women stratified by age and waiting time for KT. KT = kidney transplantation, MACE = major adverse cardiovascular event.

Stratified analysis by age and waiting time for KT showed that women had a significantly lower risk of MACEs than men in KTRs aged > 50 years (HR: 0.79; 95% CI: 0.62–1.0; *P* = .05) or with KT waiting time ≤ 6 years (HR: 0.85; 95% CI: 0.72–0.99; *P* = .04), as shown in Figure 3 and Table S2, Supplemental Digital Content, http://links.lww.com/MD/N528. However, in the other strata, the risk of MACEs was comparable in men and women.

Moreover, waiting time for KT, age, history of diabetes, and AMI were independently associated with the risk of MACEs in KTRs (*P* for all < 0.05; Figures S3 and S4, Supplemental Digital Content, http://links.lww.com/MD/N528). Similarly, the results were consistent before and after PSM.

### 3.3. BN analysis of study outcomes

**Figure 4.**
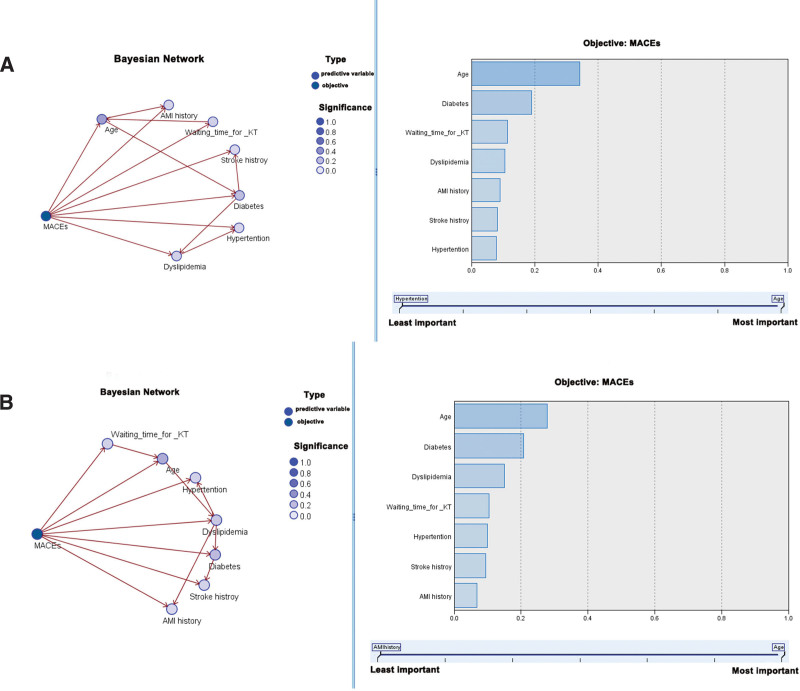
Bayesian Network of MACEs for men (A) and women (B). MACE = major adverse cardiovascular event.

In the BN model with full baseline characteristics included, we analyzed the gender differences in the effect of each covariate on MACEs separately. The BN results showed that in either in men or women, age was an important covariate for the incidence of MACEs in KTRs, whereas the importance of other factors was comparable (Fig. 4). Moreover, the importance of different covariates on MACEs remained different among men and women (Fig. 4). Meanwhile, age remains an important predictor of the incidence of MACEs in the overall population (Figure S5, Supplemental Digital Content, http://links.lww.com/MD/N528).

## 4. Discussion

The findings of this retrospective population-based cohort study can be summarized as follows: Among KTRs aged > 50 years or who had waited ≤ 6 years, the risk of MACEs and nonfatal MI was significantly lower in women than in men; in both men and women, age is an important determinant of cardiovascular outcomes in KTRs.

KT is the treatment of choice for patients with ESRD and is strongly associated with improved prognosis and reduced mortality.^[15]^ The survival benefit after KT was mainly attributable to a reduced cardiovascular disease burden, but KTRs remain at a higher risk of cardiovascular disease-related morbidity and mortality compared to the general population.^[16,17]^ Therefore, early identification of potential risk factors may be beneficial to reducing the risk of adverse cardiovascular events in KTRs. In the present study, it is observed that gender, age, waiting time for KT, diabetes, and history of AMI were closely related to adverse cardiovascular events in KTRs, which is in line with previous findings that diabetes and prior cardiovascular disease are the independent risk factors for adverse cardiovascular events in KTRs.^[12,18]^ In addition, consistent results were obtained in the BN models, suggesting that age was the most important predictor of MACEs for KTRs, which was similar to the results given by Pilmore et al^[4]^ and Lentine et al.^19]^

Currently, studies on gender differences in cardiovascular outcomes of KTRs are still limited. Oien et al^[20]^ pointed out that there was no gender difference in cardiac events or total mortality in KTRs. In their study, the primary endpoint was defined as cardiovascular death and nonfatal MI, and the secondary endpoint was all-cause death. The difference in the primary endpoint may be an important reason for the difference in outcomes, while the results for all-cause mortality are consistent with our study. Besides, the study conducted by Chen et al^[21]^ showed that men were an independent factor in the poor prognosis of KTRs. Similarly, the study by Seoane-Pillado et al^[12]^ revealed that men were a risk factor for adverse cardiovascular events, which is in line with our findings. The mechanisms behind gender differences in MACEs are still unclear and may be related to the following aspects. First, women are more likely to comply with follow-up assessments and immunosuppressive medication regimens than men, which stems from sociocultural differences between men and women. In this cultural difference, women are believed to be more aware of their health status and tend to follow the advice of their health care providers.^[22]^ Second, estrogen has a protective effect against ischemia and delays the graft function incidence.^[23]^ Finally, it has been shown that the X chromosome in women is strongly associated with KT outcomes.^[23]^ In addition, the biological sex of KTRs affects the pharmacokinetics and pharmacodynamics of immunosuppressive drugs. Affecting pharmacodynamics of a drug subsequently affect pharmacological effects and therapeutic outcomes.^[24]^

Additionally, age-stratified analysis showed that men had an increased risk of MACEs among KTRs aged > 50 years. However, in other age strata, the risk of MACEs was comparable in men and women. Similarly, among KTRs who waited ≤ 6 years for KT, men had a higher risk of MACEs, presumably related to the fact that a long wait reduced the benefit of KT. When waiting for KT, ERSD patients typically require dialysis treatment to survive. Prolonged dialysis may cause more complications, significantly reduce the benefit of kidney transplantation, and reduce the impact of gender differences on MACEs.

### 4.1. Perspectives

At present, kidney donation is still very rare and for ESRD patients who can undergo KT, it is a second “rebirth.” Improving the survival and quality of life of patients after surgery is an important consideration in preoperative evaluation. The results of this study suggest that men are at higher risk of adverse cardiovascular outcomes, and this difference is more pronounced in KTRs aged > 50 years and those waiting ≤ 6 years for KT. Therefore, men as a high-risk group require additional attention postoperatively. Furthermore, age is an important factor influencing cardiovascular outcomes in KTRs. These findings provide some new insights into the prognostic evaluation of ESRD patients and postoperative follow-up.

### 4.2. Strengths and limitations

This study has the following advantages. First, in this study, a comprehensive analysis of the risk factors for adverse cardiovascular prognosis and gender differences in KTRs was made, which might provide some guidance for future clinical studies and early prevention. Second, the study population of this paper was KTRs without complications, and the secondary analysis was conducted based on PSM, which noticeably reduced the influence of the results due to the differences in baseline characteristics. Moreover, the original cohort study was performed as a sensitivity analysis, and the results of both cohorts were consistent. Finally, previous studies of KT have mainly originated from European and American countries, and this study has enriched the knowledge of Asian people. Inevitably, the following limitations of this study should be disclosed. First, this study is a secondary analysis, and some important clinical characteristics of KTRs, such as the etiology of ERSD, the type of kidney pathology, medication use, lifestyle, and some clinical characteristics of kidney donors, are missing due to the limitations of the original study data. These factors may have some influence on the results of this study. In addition, the primary outcome of this study was MACE, including all-cause mortality. The cause of death could not be clarified because data on cardiovascular mortality could not be further obtained. However, previous studies have shown that cardiovascular mortality is the leading cause of death in patients with renal failure.^[25,26]^ Therefore, including all-cause mortality in the definition of MACE is unlikely to weaken the results we observed.

## 5. Conclusions

In Taiwan, women had a lower risk of adverse cardiovascular outcomes than men in KTRs aged > 50 years or with a waiting time for KT ≤ 6 years. Furthermore, age is an important independent determinant for the prognosis of KTR.

## Acknowledgments

We thank the authors of the original study (DOI:10.1136/bmjopen-2021-058033) for their valuable contributions and for dedicating their work to the public domain, making this research possible.

## Author contributions

**Conceptualization:** Jiang Liu, Siwei Chen, Wenqiang Gao.

**Data curation:** Jiang Liu, Siwei Chen, Wenqiang Gao.

**Formal analysis:** Jiang Liu.

**Writing – original draft:** Jiang Liu, Siwei Chen, Wenqiang Gao.

**Writing – review and editing:** Jiang Liu, Siwei Chen, Wenqiang Gao.

**Software:** Siwei Chen.

**Methodology:** Wenqiang Gao.

**Project administration:** Wenqiang Gao.

**Resources:** Wenqiang Gao.

**Supervision:** Wenqiang Gao.

**Validation:** Wenqiang Gao.

**Visualization:** Wenqiang Gao.

## Supplementary Material



## References

[1] Jha V Garcia-Garcia G Iseki K. Chronic kidney disease: global dimension and perspectives. Lancet. 2013;382:260–72. 23727169 10.1016/S0140-6736(13)60687-X

[2] Chen H-H Chern Y-B Hsu C-Y Tang P-L Lai C-C. Kidney transplantation waiting times and risk of cardiovascular events and mortality: a retrospective observational cohort study in Taiwan. BMJ Open. 2022;12:e058033. 10.1136/bmjopen-2021-058033 PMC9131177 35613763

[3] Saran R Robinson B Abbott KC. US Renal Data System 2018 annual data report: epidemiology of kidney disease in the United States. Am J Kidney Dis. 2019;73(3 Suppl 1):A7–8. 30798791 10.1053/j.ajkd.2019.01.001 PMC6620109

[4] Pilmore H Dent H Chang S McDonald SP Chadban SJ. Reduction in cardiovascular death after kidney transplantation. Transplantation. 2010;89:851–7. 20048695 10.1097/TP.0b013e3181caeead

[5] Hart A Lentine KL Smith JM. OPTN/SRTR 2019 Annual Data Report: kidney. Am J Transplant. 2021;21(Suppl 2):21–137. 10.1111/ajt.16502 33595191

[6] Wheeler DC Steiger J. Evolution and etiology of cardiovascular diseases in renal transplant recipients. Transplantation. 2000;70(11 Suppl):SS41–5. 11152230

[7] Stoumpos S Jardine AG Mark PB. Cardiovascular morbidity and mortality after kidney transplantation. Transpl Int. 2015;28:10–21. 25081992 10.1111/tri.12413

[8] de Mattos AM Prather J Olyaei AJ. Cardiovascular events following renal transplantation: role of traditional and transplant-specific risk factors. Kidney Int. 2006;70:757–64. 16788687 10.1038/sj.ki.5001628

[9] Barn K Laftavi M Pierce D Ying C Boden WE Pankewycz O. Low levels of high-density lipoprotein cholesterol: an independent risk factor for late adverse cardiovascular events in renal transplant recipients. Transpl Int. 2010;23:574–9. 20003032 10.1111/j.1432-2277.2009.01021.x

[10] Ribic CM Holland D Howell J. Study of cardiovascular outcomes in renal transplantation: a prospective, multicenter study to determine the incidence of cardiovascular events in renal transplant recipients in Ontario, Canada. Can J Kidney Health Dis. 2017;4:2054358117713729. 28660072 10.1177/2054358117713729 PMC5476328

[11] Veroux M Grosso G Corona D. Age is an important predictor of kidney transplantation outcome. Nephrol Dial Transplant. 2012;27:1663–71. 21926404 10.1093/ndt/gfr524

[12] Seoane-Pillado MT Pita-Fernández S Valdés-Cañedo F. Incidence of cardiovascular events and associated risk factors in kidney transplant patients: a competing risks survival analysis. BMC Cardiovasc Disord. 2017;17:72. 28270107 10.1186/s12872-017-0505-6 PMC5341360

[13] Chien P-L Liu C-F Huang H-T. Application of Artificial Intelligence in the establishment of an association model between metabolic syndrome, TCM constitution, and the guidance of medicated diet care. Evid Based Complement Alternat Med. 2021;2021:5530717. 34007288 10.1155/2021/5530717 PMC8110390

[14] Guo S He J Li J Tang B. Exploring the impact of unsafe behaviors on building construction accidents using a bayesian network. Int J Environ Res Public Health. 2019;17:221. 31892270 10.3390/ijerph17010221 PMC6981992

[15] Wolfe RA Ashby VB Milford EL. Comparison of mortality in all patients on dialysis, patients on dialysis awaiting transplantation, and recipients of a first cadaveric transplant. N Engl J Med. 1999;341:1725–30. 10580071 10.1056/NEJM199912023412303

[16] Anavekar NS McMurray JJV Velazquez EJ. Relation between renal dysfunction and cardiovascular outcomes after myocardial infarction. N Engl J Med. 2004;351:1285–95. 15385655 10.1056/NEJMoa041365

[17] Rangaswami J Mathew RO Parasuraman R. Cardiovascular disease in the kidney transplant recipient: epidemiology, diagnosis and management strategies. Nephrol Dial Transplant. 2019;34:760–73. 30984976 10.1093/ndt/gfz053

[18] Cosio FG Hickson LJ Griffin MD Stegall MD Kudva Y. Patient survival and cardiovascular risk after kidney transplantation: the challenge of diabetes. Am J Transplant. 2008;8:593–9. 18294155 10.1111/j.1600-6143.2007.02101.x

[19] Lentine KL Brennan DC Schnitzler MA. Incidence and predictors of myocardial infarction after kidney transplantation. J Am Soc Nephrol. 2005;16:496–506. 15615820 10.1681/ASN.2004070580

[20] Oien CM Reisaeter AV Os I Jardine A Fellström B Holdaas H. Gender-associated risk factors for cardiac end points and total mortality after renal transplantation: post hoc analysis of the ALERT study. Clin Transplant. 2006;20:374–82. 16824157 10.1111/j.1399-0012.2006.00496.x

[21] Chen P-D Tsai M-K Lee C-Y. Gender differences in renal transplant graft survival. J Formos Med Assoc. 2013;112:783–8. 24246256 10.1016/j.jfma.2013.10.011

[22] Katz-Greenberg G Shah S. Sex and gender differences in kidney transplantation. Semin Nephrol. 2022;42:219–29. 35718368 10.1016/j.semnephrol.2022.04.011 PMC10065984

[23] Aufhauser DD JR Wang Z Murken DR. Improved renal ischemia tolerance in females influences kidney transplantation outcomes. J Clin Invest. 2016;126:1968–77. 27088798 10.1172/JCI84712 PMC4855926

[24] Pan H Yang S Wang Y Shubhra QTH. Ferroptosis-based image-guided chemotherapy. Matter. 2023;6:666.

[25] Ramesh S Zalucky A Hemmelgarn BR. Incidence of sudden cardiac death in adults with end-stage renal disease: a systematic review and meta-analysis. BMC Nephrol. 2016;17:78. 27401469 10.1186/s12882-016-0293-8 PMC4940956

[26] Di Lullo L House A Gorini A Santoboni A Russo D Ronco C. Chronic kidney disease and cardiovascular complications. Heart Fail Rev. 2015;20:259–72. 25344016 10.1007/s10741-014-9460-9

