# Supplementary material for: Gender differences in cardiovascular outcomes of kidney transplant recipients: A retrospective cohort study
Source: Medicine (Baltimore). 2024 Sep 13;103(37):e39568. doi: 10.1097/MD.0000000000039568 (PMC11404969; doi:10.1097/MD.0000000000039568)
Supplement: Supplementary file 1 [file medi-103-e39568-s001.pdf]

Supplementary table 1. Proportional hazards assumption.

Supplementary Table 2. Multivariate Cox regression analysis of MACE for men and women stratified by age and waiting time for KT.

Supplementary Figure 1A: Data distribution of the original cohort according to propensity score.

Supplementary Figure 1B: Data distribution of the matched cohort according to propensity score.

Supplementary Figure 2: Kaplan-Meier analysis of original cohort.

Supplementary Figure 3: Univariate and multivariate Cox analysis of MACEs in the matched cohort.

Supplementary Figure 4: Univariate and multivariate COX analysis of MACE in the original cohort.

Supplementary Figure 5. Bayesian Network of MACEs for the overall population. MACEs, major adverse cardiovascular events.

Supplementary table 1. Proportional hazards assumption

	After PSM	Before PSM
	p-value*	p-value*
MACE	0.324	0.342
All-cause death	0.283	0.205
Non-fatal MI	0.830	0.965
Non-fatal Stroke	0.119	0.167

***P-values were tested by Schoenfeld residuals. PSM, propensity score matching. MACE, major adverse cardiovascular events.**

Supplementary Table 2. Multivariate Cox regression analysis of MACE for men and women stratified by age and waiting time for KT

	After PSM			Before PSM		
	n	HR	p-value	n	HR	p-value
Age (years)						
18-30	428	0.91 (0.50-1.70)	0.80	499	0.83 (0.509-1.567)	0.69
30-40	718	0.93 (0.64-1.37)	0.72	854	1.0 (0.70-1.42)	0.99
40-50	981	0.80 (0.61-1.04)	0.10	1196	0.80 (0.63-1.02)	0.07
≤50	2127	0.88 (0.71-1.08)	0.21	2549	0.89 (0.74-1.07)	0.22
＞50	777	0.79 (0.62-1.0)	0.05	1013	0.79 (0.64-0.98)	0.03
Waiting time for KT (years)						
＜1	690	0.96 (0.72-1.29)	0.90	853	0.93 (0.72-1.21)	0.60
1-3	1359	0.78 (0.62-0.98)	0.03	1652	0.85(0.69-1.04)	0.12
4-6	603	0.74 (0.51-1.08)	0.18	750	0.68 (0.49-0.94)	0.02
≤6	2652	0.85 (0.72-0.99)	0.04	3255	0.85 (0.73-0.98)	0.02
＞6	252	0.79 (0.41-1.66)	0.63	307	0.94 (0.52-1.70)	0.85

PSM, propensity score matching. MACE, Major adverse cardiovascular events. KT, Kidney transplantation.

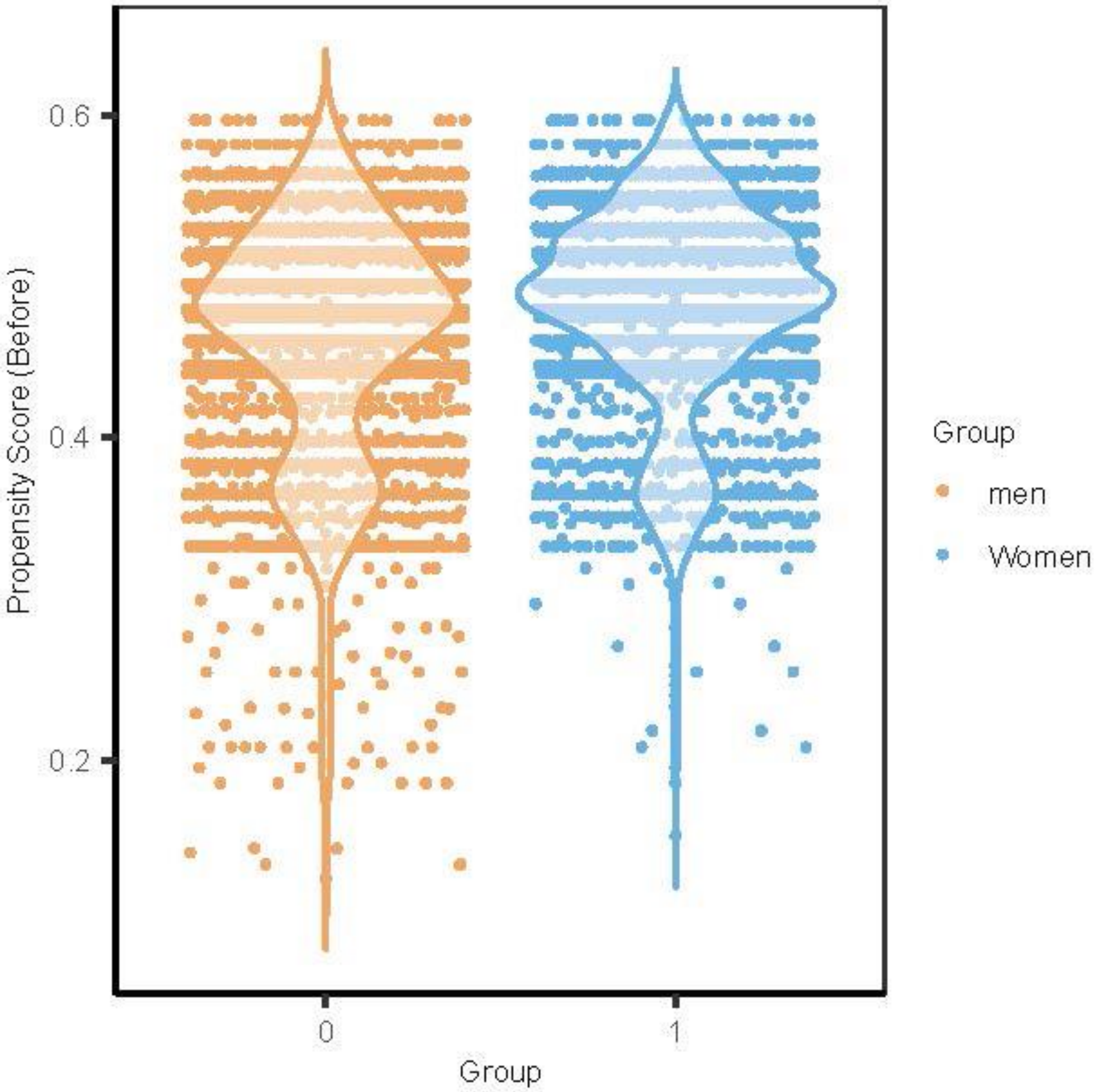

Propensity Score (Before)
0.6
0.4
0.2
0
1
Group
Group
men
Women

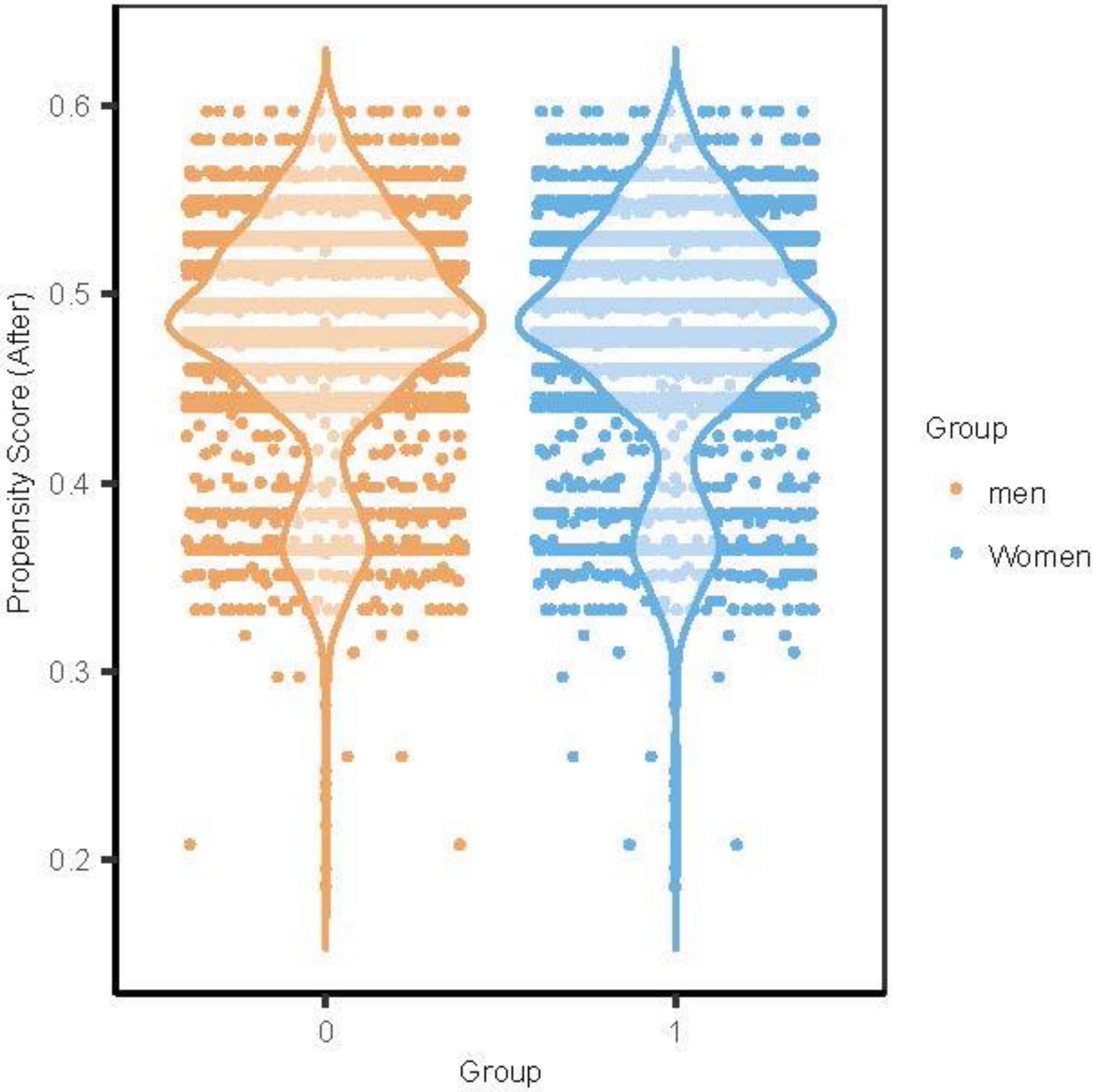

0.6
0.5
0.4
0.3
0.2
Propensity Score (After)
0
1
Group
Group
men
Women

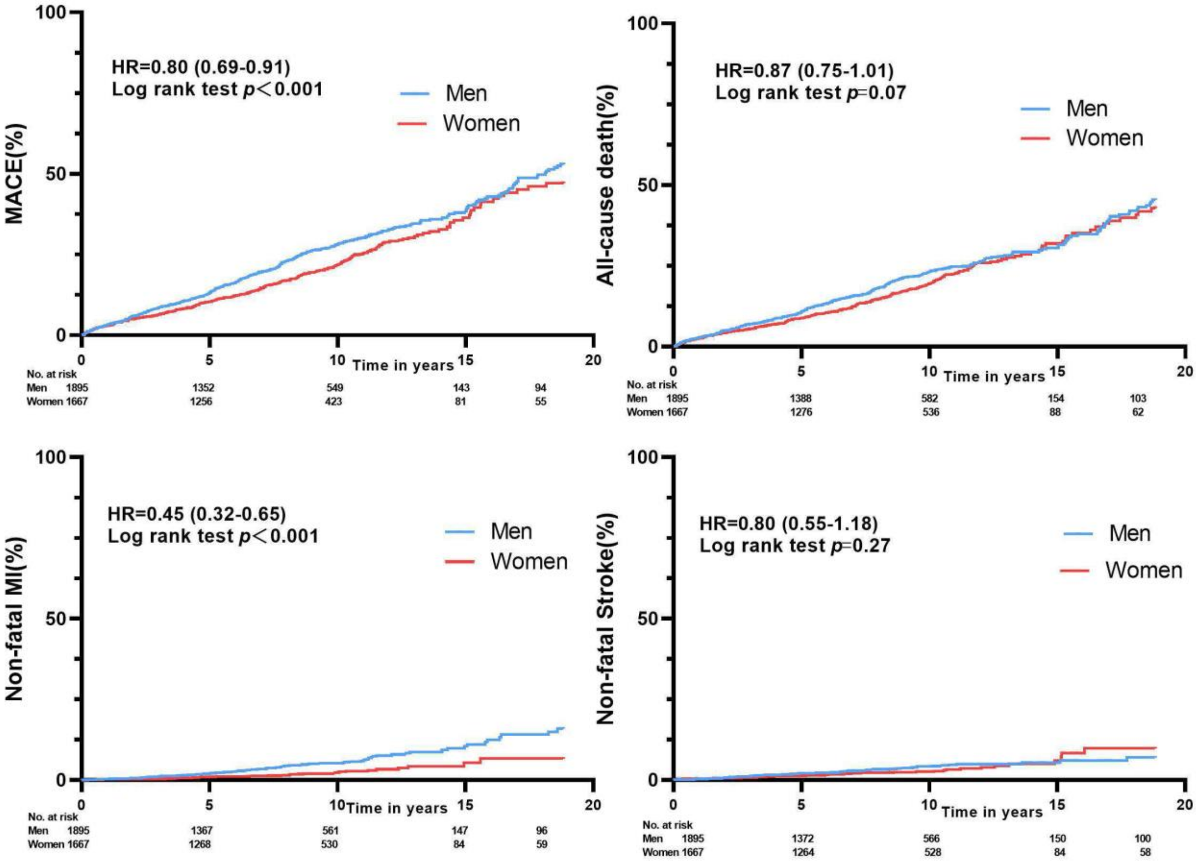

MACE(%)
HR=0.80 (0.69-0.91)
Log rank test p＜0.001
Men
Women
Time in years
No. at risk
Men 1895 1352 549 143 94
Women 1667 1256 423 81 55
All-cause death(%)
HR=0.87 (0.75-1.01)
Log rank test p=0.07
Men
Women
Time in years
No. at risk
Men 1895 1388 582 154 103
Women 1667 1276 536 88 62
Non-fatal MI(%)
HR=0.45 (0.32-0.65)
Log rank test p＜0.001
Men
Women
Time in years
No. at risk
Men 1895 1367 561 147 96
Women 1667 1268 530 84 59
Non-fatal Stroke(%)
HR=0.80 (0.55-1.18)
Log rank test p=0.27
Men
Women
Time in years
No. at risk
Men 1895 1372 566 150 100
Women 1667 1264 528 84 58

Characteristics	Number (%)	Univariate Cox Analysis HR (95%CI)	Univariate P-value	Multivariate Cox Analysis HR (95%CI)	Multivariate P-value
**Sex**					
Men	1452 (50.0)				
Women	1452 (50.0)	0.84 [0.72, 0.98]	0.024	0.82 [0.70, 0.95]	0.011
**Waiting time for KT**					
<1 year	690 (23.8)				
>6 years	252 (8.7)	1.88 [1.29, 2.73]	0.001	2.97 [2.02, 4.36]	<0.001
4-6 years	603 (20.8)	1.69 [1.32, 2.18]	<0.001	2.19 [1.69, 2.84]	<0.001
1-3 years	1359 (46.8)	1.51 [1.23, 1.85]	<0.001	1.79 [1.46, 2.20]	<0.001
**Age**					
≤40 years	1146 (39.5)				
>60 years	144 (5.0)	4.45 [3.34, 5.93]	<0.001	4.52 [3.33, 6.14]	<0.001
40-60 years	1614 (55.6)	2.22 [1.84, 2.68]	<0.001	2.27 [1.87, 2.75]	<0.001
**Diabetes**					
No	2404 (82.8)				
Yes	500 (17.2)	2.00 [1.67, 2.39]	<0.001	1.71 [1.40, 2.08]	<0.001
**Hypertension**					
No	708 (24.4)				
Yes	2196 (75.6)	1.15 [0.96, 1.37]	0.136	0.96 [0.79, 1.16]	0.652
**Dyslipidemia**					
No	2188 (75.3)				
Yes	716 (24.7)	1.28 [1.07, 1.53]	0.006	0.97 [0.79, 1.18]	0.751
**History of AMI**					
No	2875 (99.0)				
Yes	29 (1.0)	3.01 [1.70, 5.32]	<0.001	1.88 [1.05, 3.36]	0.033
**History of stroke**					
No	2881 (99.2)				
Yes	23 (0.8)	2.45 [1.35, 4.44]	0.003	1.33 [0.72, 2.45]	0.362

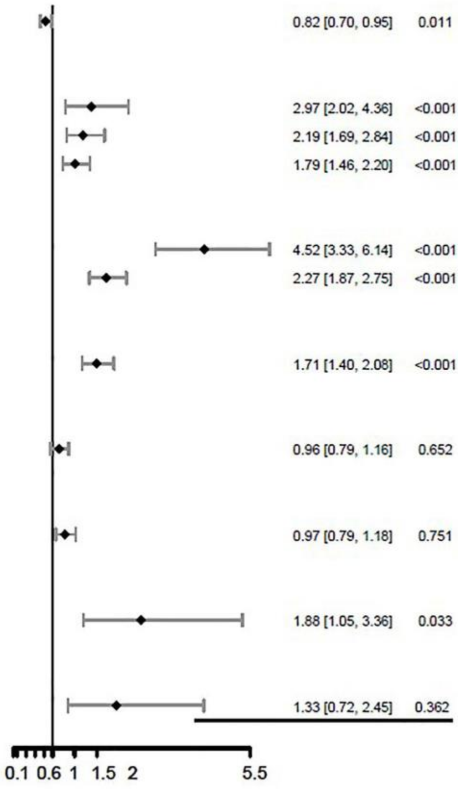

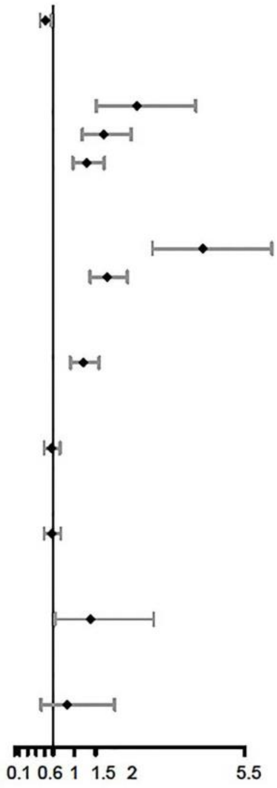

Univariate COX Analysis | **Multivariate COX Analysis**

Characteristics	Number (%)	HR (95%CI)	P-value	HR (95%CI)	P-value
**Sex**					
Men	1895 (53.2)				
Women	1667 (46.8)	0.79 [0.69, 0.91]	0.001	0.82 [0.71, 0.94]	0.006
**Waiting time for KT**					
＜1 year	853 (23.9)				
＞6 years	307 (8.6)	1.81 [1.30, 2.51]	<0.001	2.92 [2.08, 4.09]	<0.001
4-6 years	750 (21.1)	1.64 [1.32, 2.04]	<0.001	2.12 [1.70, 2.65]	<0.001
1-3 years	1652 (46.4)	1.41 [1.19, 1.68]	<0.001	1.64 [1.37, 1.95]	<0.001
**Age**					
≤40 years	1353 (38.0)				
＞60 years	225 (6.3)	4.69 [3.70, 5.94]	<0.001	4.32 [3.36, 5.55]	<0.001
40-60 years	1984 (55.7)	2.32 [1.96, 2.75]	<0.001	2.25 [1.89, 2.68]	<0.001
**Diabetes**					
No	2803 (78.7)				
Yes	759 (21.3)	2.13 [1.84, 2.46]	<0.001	1.67 [1.41, 1.97]	<0.001
**Hypertension**					
No	828 (23.2)				
Yes	2734 (76.8)	1.21 [1.03, 1.42]	0.022	0.94 [0.79, 1.12]	0.505
**Dyslipidemia**					
No	2587 (72.6)				
Yes	975 (27.4)	1.30 [1.12, 1.51]	0.001	0.98 [0.83, 1.16]	0.852
**History of AMI**					
No	3486 (97.9)				
Yes	76 (2.1)	2.57 [1.81, 3.64]	<0.001	1.52 [1.07, 2.17]	0.021
**History of stroke**					
No	3491 (98.0)				
Yes	71 (2.0)	3.26 [2.37, 4.48]	<0.001	1.95 [1.40, 2.72]	<0.001

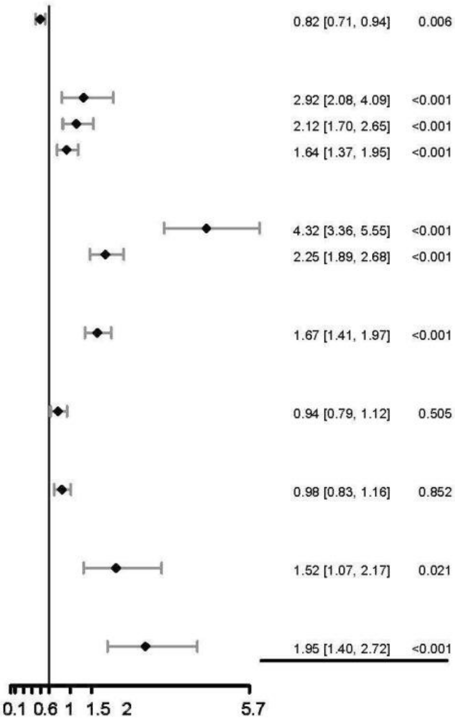

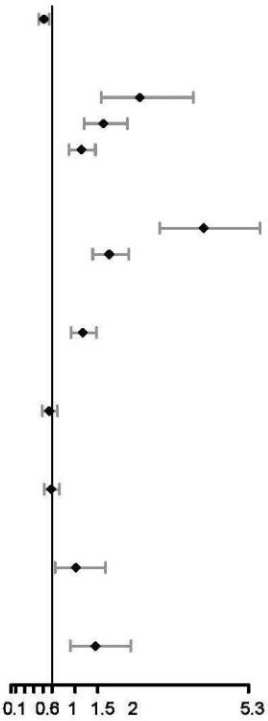

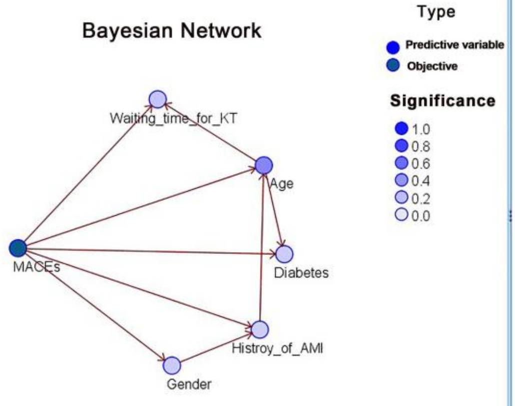
Bayesian Network
Waiting_time_for_KT
Age
MACEs
Diabetes
Histroy_of_AMI
Gender
Type
Predictive variable
Objective
Significance
1.0
0.8
0.6
0.4
0.2
0.0

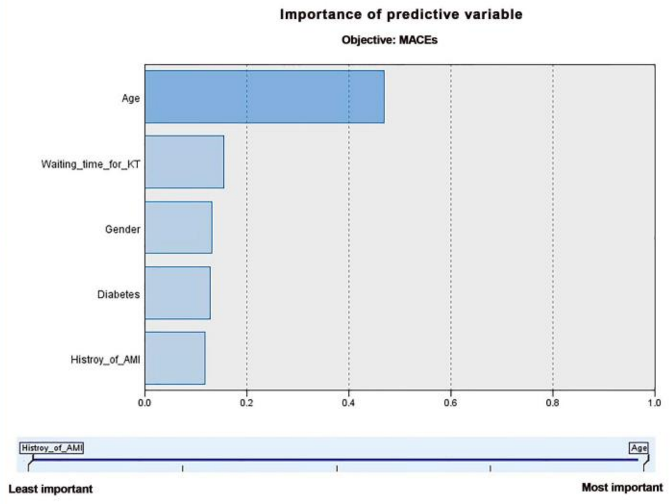
Importance of predictive variable
Objective: MACEs
Age
Waiting_time_for_KT
Gender
Diabetes
Histroy_of_AMI
0.0
0.2
0.4
0.6
0.8
1.0
Histroy_of_AMI
Age
Least important
Most important
